# Identification of cholinergic centro-cingulate topography as main contributor to cognitive functioning in Parkinson’s disease: Results from a data-driven approach

**DOI:** 10.3389/fnagi.2022.1006567

**Published:** 2022-10-20

**Authors:** Sygrid van der Zee, Prabesh Kanel, Martijn L. T. M. Müller, Teus van Laar, Nicolaas I. Bohnen

**Affiliations:** ^1^Department of Radiology, University of Michigan, Ann Arbor, MI, United States; ^2^Department of Neurology, University of Groningen, University Medical Center Groningen, Groningen, Netherlands; ^3^Morris K. Udall Center of Excellence for Parkinson’s Disease Research, University of Michigan, Ann Arbor, MI, United States; ^4^Department of Neurology, University of Michigan, Ann Arbor, MI, United States; ^5^Neurology Service and Geriatric Research Education and Clinical Center (GRECC), Veterans Administration Ann Arbor Healthcare System, Ann Arbor, MI, United States; ^6^University of Michigan Parkinson’s Foundation Center of Excellence, Ann Arbor, MI, United States

**Keywords:** Parkinson’s disease, cognition, acetylcholine, PET, neuroimaging

## Abstract

**Background:**

Degeneration of the cholinergic system plays an important role in cognitive impairment in Parkinson’s disease (PD). Positron emission tomography (PET) imaging using the presynaptic vesicular acetylcholine transporter (VAChT) tracer [^18^F]Fluoroethoxybenzovesamicol ([^18^F]FEOBV) allows for regional assessment of cholinergic innervation. The purpose of this study was to perform a data-driven analysis to identify co-varying cholinergic regions and to evaluate the relationship of these with cognitive functioning in PD.

**Materials and methods:**

A total of 87 non-demented PD patients (77% male, mean age 67.9 ± 7.6 years, disease duration 5.8 ± 4.6 years) and 27 healthy control (HC) subjects underwent [^18^F]FEOBV brain PET imaging and neuropsychological assessment. A volume-of-interest based factor analysis was performed for both groups to identify cholinergic principal components (PCs).

**Results:**

Seven main PCs were identified for the PD group: (1) bilateral posterior cortex, (2) bilateral subcortical, (3) bilateral centro-cingulate, (4) bilateral frontal, (5) right-sided fronto-temporal, (6) cerebellum, and (7) predominantly left sided temporal regions. A complementary principal component analysis (PCA) analysis in the control group showed substantially different cholinergic covarying patterns. A multivariate linear regression analyses demonstrated PC3, PC5, and PC7, together with motor impairment score, as significant predictors for cognitive functioning in PD. PC3 showed most robust correlations with cognitive functioning (*p* < 0.001).

**Conclusion:**

A data-driven approach identified covarying regions in the bilateral peri-central and cingulum cortex as a key determinant of cognitive impairment in PD. Cholinergic vulnerability of the centro-cingulate network appears to be disease-specific for PD rather than being age-related. The cholinergic system may be an important contributor to regional and large scale neural networks involved in cognitive functioning.

## Introduction

Parkinson’s disease (PD) is best known as a movement disorder related to dopaminergic degeneration of the substantia nigra pars compacta. However, non-motor symptoms, including cognitive impairment, are common in PD and contribute significantly to lower quality of life (Post et al., 2011; Lawson et al., 2014). Degeneration of the cholinergic system has been implicated as a significant contributor to both motor and non-motor symptoms in PD, including postural instability and gait disorder, cognition, mood, olfaction, and rapid eye movement (REM) sleep behavior disorder (Bohnen and Albin, 2011; Müller and Bohnen, 2013; Halliday et al., 2014).

The cholinergic system includes widespread projections throughout the brain, originating from four major sources: (1) the basal forebrain cholinergic cell group (including Ch1-4) with widespread projections throughout the brain; (2) the pedunculopontine nucleus-laterodorsal tegmental complex (PPN/LDT, Ch5, and Ch6) projecting to the thalamus, striatum, brainstem, and cerebellum; (3) the medial vestibular nucleus (MVN) providing cholinergic input to the cerebellum; and (4) local striatal cholinergic interneurons (Mesulam et al., 1983; Zhang et al., 2016; Craig et al., 2020).

There is evidence for vulnerability of the brain cholinergic projections in PD. *In vivo* studies using acetylcholinesterase (AChE) positron emission tomography (PET) have revealed significant cholinergic denervation in a subset of non-demented PD compared to control subjects (Shimada et al., 2009; Klein et al., 2010; Bohnen et al., 2012). Cholinergic losses are even more pronounced in PD patients with dementia, suggesting a direct relationship between cognitive impairment and cholinergic denervation in PD (Hilker et al., 2005).

The exact role of cholinergic innervation in PD and subsequent cognitive impairment remains elusive. Due to widespread cholinergic projections, the cholinergic system was traditionally viewed as a diffuse modulatory system (Woolf, 1991). However, there is emerging evidence to support a more regional deterministic role of the cholinergic system (Sarter et al., 2014; Ballinger et al., 2016). We recently demonstrated a regional cholinergic denervation pattern related to multi-domain cognitive functioning in non-demented PD subjects that was derived from whole brain vesicular acetylcholine transporter (VAChT) PET voxel-based correlation analysis (van der Zee et al., 2020). However, it is unclear if the shared cognitive topographic pattern that we identified in our previous work is derived from whole brain diffuse projections or related to small to larger scale neural networks. To get a better understanding of the cholinergic system and its role in the neural networks, we aimed to evaluate the cholinergic connectivity.

The purpose of this study is to (1) perform a PET-based data-driven approach to identify covarying cholinergic brain regions, and then (2) correlate identified covarying components with cognitive performance measure in PD without dementia. To achieve this goal, we applied a factor analysis on volume of interest (VOI) based cholinergic brain PET regions. This approach was used to determine the extent to which shared variance exists between brain cholinergic regions within PD, and to characterize the regional functioning of the cholinergic system, both to a smaller and larger extent within the brain networks. Cholinergic innervation is assessed using the VAChT tracer [^18^F]Fluoroethoxybenzovesamicol ([^18^F]FEOBV). [^18^F]FEOBV PET imaging has the distinct advantage to allow a more detailed *in vivo* regional assessment of cholinergic innervation in both cortical and subcortical brain regions compared to previously used AChE PET ligands (Petrou et al., 2014; Nejad-Davarani et al., 2019; van der Zee et al., 2019). Based on previous AChE PET imaging studies in PD we hypothesized the posterior cortical regions may be driving a significant portion of the variance in cognitive data in PD.

## Materials and methods

### Participants

A total of 90 Patients with PD and 27 healthy control (HC) subjects were included in this study. Inclusion criteria for patients consisted of a clinical PD diagnosis according to the UK Parkinson’s Disease Society Brain Bank clinical diagnostic criteria (Hughes et al., 1992). Exclusion criteria were the use of anticholinergic or cholinesterase inhibitor drugs and evidence of large vessel stroke or other intracranial lesions on anatomic imaging. Three subjects were excluded due to technical difficulties, including severe motion during image acquisition. All subjects gave written informed consent. The study was approved by the Institutional Review Boards of the University of Michigan School of Medicine and Veterans Affairs Ann Arbor Healthcare System. Data included in this study has previously been published in our study on the whole brain topographic cholinergic correlates of cognitive domain functioning (van der Zee et al., 2020).

### Clinical assessment

All PD patients underwent a detailed neuropsychological assessment covering the main cognitive domains: memory, attention, executive function, language, and visuospatial abilities. The assessment included at least two neuropsychological tests for each cognitive domain, in line with Movement Disorder Society (MDS) Task force criteria for the assessment of mild cognitive impairment in Parkinson’s disease with mild cognitive impairment (PD-MCI) (Litvan et al., 2012; Goldman et al., 2015). The neuropsychological test battery has previously been described (van der Zee et al., 2020), and included the California Verbal Learning Test (CVLT) and Wechsler Memory Scale (WMS) for the assessment of memory; the Stroop color test, Delis-Kaplan Executive Function System (DKEFS) Trail Making Test (TMT) 2: number sequencing, and the Wechsler Adult Intelligence Scale (WAIS) Digit Span (DS) backward for the assessment of the attention domain; the Stroop Adjusted Color Word test, DKEFS TMT 4: letter-number sequencing, the WAIS matrix reasoning task and the letter fluency task for the assessment of executive functions; the verbal fluency (animals) and the Boston Naming Test (BNT) to assess the language domain; and the Benton Judgment of Line Orientation (JOLO) and the Clock Copy Test of the PD-Cognitive Rating scale (Pagonabarraga et al., 2008) for the assessment of visuospatial function. Neuropsychological testing was performed in the patients while they were on their usual dopaminergic medications.

In addition, all patients underwent motor assessment using the Movement Disorder Society-Revised Unified Parkinson’s Disease Rating Scale part III (MDS-UPDRS-III). Motor assessment was performed in the dopaminergic medication “off” state, after withdrawal of dopaminergic medication overnight.

### Imaging techniques and analysis

Imaging techniques were applied as described previously (Bohnen et al., 2019; van der Zee et al., 2020). In short, all subjects underwent brain MRI and VAChT [^18^F]FEOBV PET imaging. T1-weighted MRI was performed on a 3 Tesla Philips Achieva system (Philips, Best, Netherlands). A 3D inversion recovery-prepared turbo-field-echo was performed in the sagittal plane using TR/TE/TI = 9.8/4.6/1,041 ms; turbo factor = 200; single average; field-of-view = 240 × 200 × 160 mm; acquired Matrix = 240 × 200 × 160 slices and reconstructed to 1 mm isotropic resolution. PET imaging was performed in 3D imaging mode with an emission computerised axial tomography (ECAT) Exact HR + tomograph (Siemens Molecular Imaging, Inc., Knoxville, TN, United States), which acquires 63 transaxial slices [slice thickness: 2.4 mm; intrinsic in-plane resolution: 4.1 mm full-width at half maximum (FWHM) over a 15.2 cm axial field-of-view].

[^18^F]FEOBV was prepared as described previously (Shao et al., 2011b,a). [^18^F]FEOBV delayed dynamic imaging was performed over 30 min (in six 5-min frames) starting 3 h after an intravenous bolus dose injection of 8 mCi [^18^F]FEOBV (Petrou et al., 2014). The PET imaging frames were spatially coregistered within subjects with a rigid-body transformation to reduce the effects of subject motion during the imaging session (Minoshima et al., 1993). Statistical parametric mapping (SPM) software (SPM12; Wellcome Trust Centre for Neuroimaging, University College, London, England) was used for PET-MRI registration. Freesurfer software^1^ was used to define cortical and subcortical MR gray VOI. A white matter reference tissue approach was used to determine VAChT binding as previously validated (Aghourian et al., 2017; Nejad-Davarani et al., 2019). Supratentorial white matter above the lateral ventricles was used to account for the partial volume effect that might arise from the high binding region (the striatum and thalamus). An erosion to the supratentorial white matter mask was performed to minimize the partial volume effects from the cortical areas. Distribution volume ratios (DVR) were calculated from ratio of averaged frames for gray matter target and white matter reference tissues. We computed subject DVR at VOI as a ratio of mean activity withing the region and subjects’ supratentorial white matter. We used a locally develop application for computing DVR. VOIs were defined based on the Mindboggle-101 dataset segmented in FreeSurfer. Since the time activity curve at our reference region reaches an equilibrium starting at 3 h post-injection, the DVR yield in our study using the method described above is comparable to the standardized uptake value ratio (SUVr) value used previously (Aghourian et al., 2017).

### Statistical analysis

A *z*-score for every subject on each cognitive test was calculated based on a dataset of a healthy elderly control (HC) group of 77 subjects of similar age, gender and educational level distribution (van der Zee et al., 2020). All *z*-scores within one cognitive domain were averaged to define domain-specific *z*-score for each of the five domains. The average of the five-domain *z*-scores was used as *z*-score for overall cognitive functioning.

A factor analysis with principal component analysis (PCA) method was performed for the PD patients and HC separately in order to characterize group specific components. The analysis included all cortical and subcortical gray matter VOIs, totaling 88 VOIs, 44 for each hemisphere. A data-driven PCA approach was used to determine the extent to which shared variance exists between brain cholinergic PET VOIs in order to enact a data-reducing approach of brain regions for further exploration of the role of the cholinergic system in cognitive functioning in PD. Initially a threshold eigenvalue >1 was used to identify cholinergic principal components (PCs). Subsequently, the screeplot for explained variance was used to determine the main PCs by identifying where the curve flattened out. The varimax rotation method was applied. A loading greater than 0.4 was used to identify high binding PET regions for each component. These loadings were used to compute component scores. For each VOI, the component with the highest loading factor was selected. Using SPM, a mask was created of the VOIs with the highest loading factor for each component, to schematically visualize the PCs. After identifying the main PCs, the relationships between the cholinergic PCs and cognitive domain *z*-scores for the PD group were examined. A multivariate stepwise linear regression analysis for overall cognitive functioning was performed, with cognitive *z*-score as dependent variable and the PC scores, disease duration, MDS-UPDRS part III scores and levodopa equivalent dose (LED) (Tomlinson et al., 2010) as independent variables. Subsequently, the same multiple linear regression analysis was performed for the five-domain specific *z*-scores. Age, gender, and educational level were controlled for by the use of cognitive *z*-scores and therefore not included in the regression model. Analyses were performed using International Business Machines (IBM) Statistical Package for the Social Sciences (SPSS) Statistics for Windows, Version 24.0 (Armonk, NY, United States: IBM Corp.), statistical analysis was considered significant for α < 0.05.

## Results

### Demographic and clinical characteristics

Healthy control subjects (12 males and 15 females) had a mean (SD) age of 72.6 (7.8) years. PD subjects (67 males and 20 females) had a mean (SD) age of 67.9 (7.6) years and mean motor disease duration of 5.8 (4.6) years. Motor assessment showed a mean (SD) MDS-UPDRS-III score of 34.3 (12.1) and Hoehn and Yahr scores of 2.4 (0.6). Based on MDS PD-MCI level II criteria (Litvan et al., 2011), 39 (44.8%) of PD patients were classified as PD-MCI, 36 of which presenting with multiple-domain cognitive impairments. Table 1 gives a detailed description of the clinical and demographic characteristics.

**TABLE 1:** Demographic and clinical characteristics of included Parkinson’s disease (PD) subjects.

	*n* = 87
Age	67.9 (7.6)
Gender (m:f)	67:20
Education (years)	15.6 (2.7)
Motor disease duration (years)	5.8 (4.6)
Hoehn and Yahr stage	2.4 (0.6)
MDS-UPDRS-III	34.3 (12.1)
LED (mg)	639.7 (413)
Cognitive status (PD-NC:PD-MCI)	48:39
*Z*-score memory	−0.44 (1.03)
*Z*-score attention	−0.41 (0.87)
*Z*-score executive function	−0.55 (1.27)
*Z*-score language	−0.48 (1.11)
*Z*-score visuospatial function	−0.12 (0.84)

Mean and SD are presented for numerical variables. MDS-UPDRS, Movement Disorders Society-revised Unified Parkinson’s Disease Rating Scale; LED, Levodopa Equivalent Dose; PD-NC, Parkinson’s disease normal cognition; PD-MCI, Parkinson’s disease with mild cognitive impairment.

### Principal component analysis in Parkinson’s disease

**FIGURE 1:**
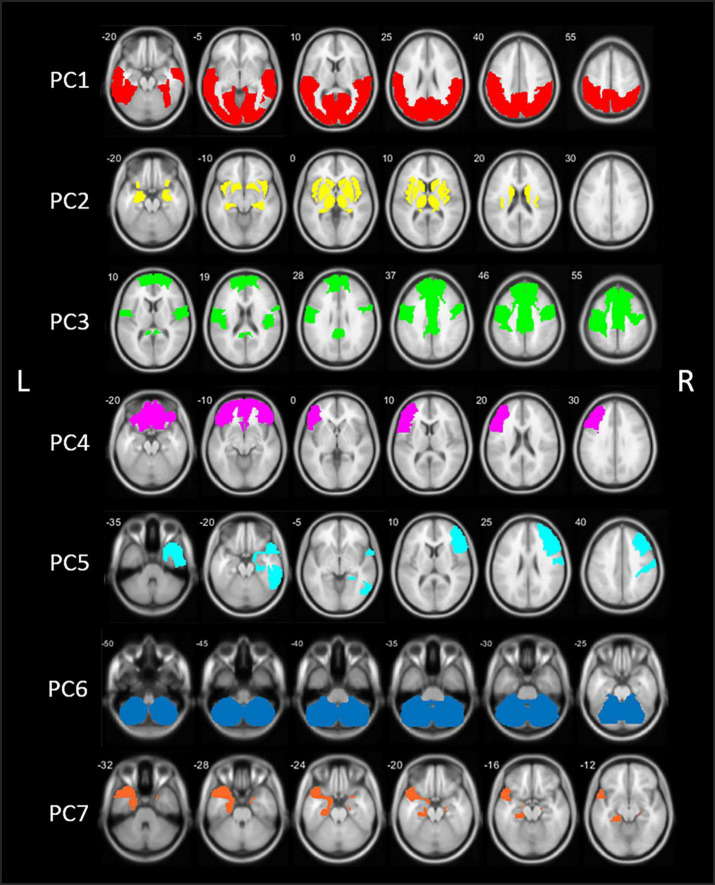
Schematic representation of the seven principal components (PCs) of the principal component analysis (PCA) in patients with Parkinson’s disease (PD). PC, Principal component; L, left; R, right.

The PCA analysis of [^18^F]FEOBV VOIs yielded 11 principle components (PCs) with an eigenvalue >1. Based on the screeplot and eigenvalues, we concentrated our analysis on the first 7 PCs, explaining 81.2% of the total variance in cholinergic binding. This reduced our dataset from 88 VOIs to 7 PCs (Supplementary Figure 1 and Table 1). A schematic representation of the first 7 components is presented in Figure 1. Loading factors for each of the seven main principal components in PD.

The first component (PC1) accounted for 47.3% of total variance in cholinergic binding and was characterized by bilateral posterior topography, comprising the occipital lobe, parietal lobe with the exemption of the postcentral gyrus, and posterior regions of the temporal lobes.

The second component (PC2) consisted of predominant subcortical and limbic topography, including the basal ganglia, thalamus, metathalamus, hippocampus, amygdala, and the bilateral insula. PC2 accounted for 14.3% of total variance. No significant correlations with cognitive scores were found.

The third component (PC3) accounted for 7.0% of total variance and was characterized by centro-cingulate regions. VOIs with highest loadings on PC3 included the per-central cortical (pre-, para-, and post-central gyri), the cingulate regions (posterior and mid to anterior regions), and the bilateral superior frontal gyri. PC3 scores were positively correlated to general cognitive functioning (*r* = 0.40, *p* < 0.001) and several cognitive domain *z*-scores, including memory (*r* = 0.47, *p* < 0.001), executive functions (*r* = 0.36, *p* < 0.001), attention (*r* = 0.36, *p* < 0.001), and language (*r* = 0.27, *p* = 0.006). For those cognitive domains, performance was worse in patients with lower PC3 scores, corresponding with lower cholinergic binding in this centro-cingulate cluster.

Components 4 and 5 showed asymmetric hemispheric covarying regions. PC4 is characterized by the frontal regions, predominantly in the left hemisphere. The component accounted for 4.3% of total variance and included the bilateral frontal pole and orbitofrontal regions, and the left pars triangularis/rostral middle frontal gyrus. PC4 did not show significant correlations with cognitive scores.

The fifth component (PC5) accounted for 3.3% of total variance and represented exclusively right-sided regions, predominantly in the frontal and temporal regions. More specific, the middle and inferior frontal gyrus, postcentral gyrus, inferior temporal lobe, and parahippocampal region showed highest loading factors on PC5. PC5 scores were positively correlated with *z*-scores on general cognition (*r* = 0.21, *p* = 0.028) and the memory domain (*r* = 0.29, *p* = 0.004), reflecting worse cognitive performance on the memory domain is associated with lower scores on PC5.

The sixth component (PC6) was characterized by the cerebellum and accounted for 2.6% of total variance. No significant correlations with the cognitive domain scores were found.

The seventh component (PC7), included the bilateral entorhinal region and the left temporal region, including the temporal pole and parahippocampal region. PC7 accounted for 2.5% of total variance. PC7 scores were positively correlates with general cognitive functioning (*r* = 0.24, *p* = 0.011), executive function (*r* = 0.25, *p* = 0.010), attention (*r* = 0.20, *p* = 0.032), and language (*r* = 0.24, *p* = 0.014).

### Principal component analysis in healthy control

**FIGURE 2:**
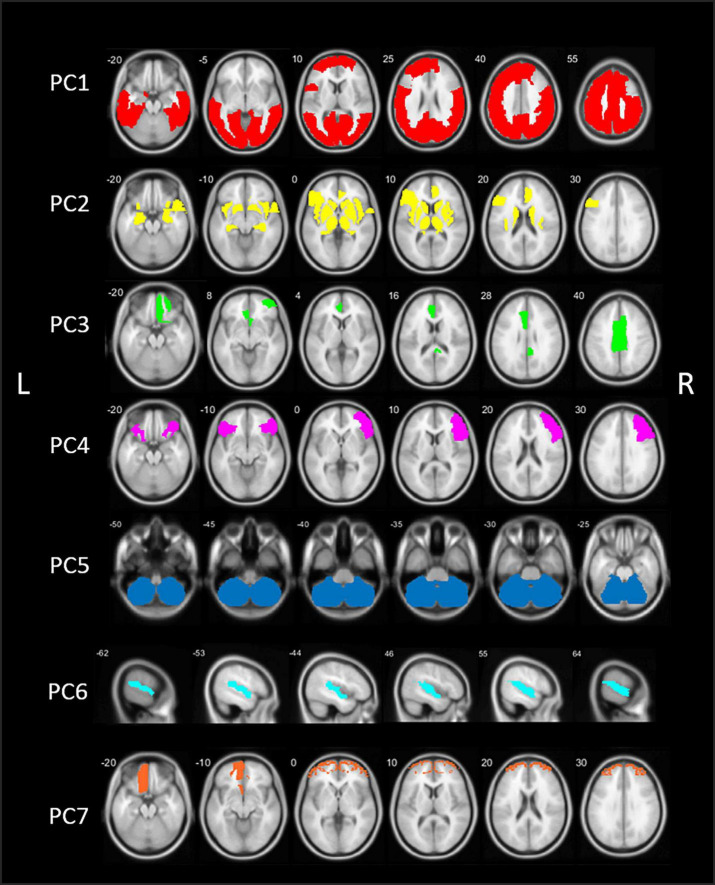
Schematic representation of the seven principal components (PCs) of the principal component analysis (PCA) in healthy control (HC) subjects. PC, Principal component; L, left; R, right.

The PCA analysis of [^18^F]FEOBV VOIs in HC yielded 12 PCs with an eigenvalue >1. Based on the screeplot (Supplementary Figure 2) and eigenvalues, three main components were identified, explaining 71.9% of total variance. For optimal comparison with the PCs resulting from the analysis on PD patients, the first 7 PCs are further described. A schematic representation of the first 7 components is presented in Figure 2.

The first component (PC1) consisted of widespread bilateral cortical regions including the occipital lobe, parietal lobe, the central regions (post-, para-, and precentral gyri), temporal regions including the middle and inferior temporal gyri, fusiform gyri and parahippocampal gyrus, and regions of the frontal lobe (superior and middle frontal gyri). PC1 accounted for 56.5% of total variance. PC2 included predominant subcortical and limbic regions, including the basal ganglia, thalamus, metathalamus, hippocampus, amygdala, and the bilateral insula. In addition, the temporal pole and entorhinal cortex were included, as well as the right lateral orbitofrontal region and left pars triangularis. PC2 accounted for 11.8% of total variance. The third component (PC3) accounted for 3.7% of total variance and consisted of the bilateral anterior cingulate and right posterior cingulate.

Components 4–7 accounted for an additional 12.3% of total variance. PC4 included the right sided pars triangularis, opercularis orbitalis, rostral middle frontal, and left pars orbitals. PC5 is characterized by the cerebellum. PC6 included the bilateral transverse temporal and superior temporal regions and PC7 consisted of the bilateral frontal pole and left orbitofrontal region. Loading factors for each of the seven main principal components of healthy controls are presented in the Supplementary Table 2.

### Multiple linear regression analysis in Parkinson’s disease

Multiple linear regression was used to identify correlates of general cognitive *z*-score based on the seven PC scores, disease duration, LED score, and MDS-UPDRS part III score. A significant regression model was found with PC3, MDS-UPDRS part III, PC7, and PC5 as significant predictors (*R*^2^ = 0.352, *p* < 0.001; Table 2).

**TABLE 2:** Regression models for general cognition and domain specific cognitive functioning.

	General cognition		Memory		Executive function		Attention		Language		Visuospatial	
	*Z*-score		*Z*-score		*Z*-score		*Z*-Score		*Z*-score		*Z*-score	
Independent												
variable	β	*p*	β	*p*	β	*p*	β	*p*	β	*p*	β	*p*
PC 1	–0.004	0.961	0.005	0.957	0.015	0.870	0.001	0.996	–0.085	0.410	0.079	0.450
PC 2	0.150	0.092	0.148	0.098	0.129	0.158	0.148	0.123	0.133	0.194	0.023	0.824
PC 3	0.325	**<0.001**	0.418	**<0.001**	0.276	**0.004**	0.256	**0.003**	0.266	**0.011**	0.041	0.700
PC 4	–0.097	0.281	–0.111	0.220	–0.048	0.603	–0.039	0.687	–0.137	0.179	–0.021	0.838
PC 5	0.197	**0.030**	0.281	**0.002**	0.124	0.176	0.083	0.390	0.152	0.138	0.152	0.141
PC 6	–0.126	0.159	–0.022	0.809	–0.128	0.163	–0.180	**0.029**	–0.042	0.682	–0.115	0.269
PC 7	0.240	**0.008**	0.111	0.215	0.243	**0.009**	0.168	**0.040**	0.236	**0.023**	0.161	0.119
Disease duration	–0.052	0.587	–0.036	0.708	0.045	0.641	0.094	0.258	–0.129	0.225	–0.128	0.230
MDS-UPDRS-III	–0.312	**0.001**	–0.202	**0.031**	–0.358	**<0.001**	–0.020	**0.004**	–0.186	0.073	–0.315	**0.003**
LED	–0.108	0.265	–0.064	0.508	–0.057	0.565	–0.009	0.919	–0.140	0.183	–0.103	0.352
	*R*^2^: 0.352		*R*^2^: 0.336		*R*^2^: 0.312		*R*^2^: 0.282		*R*^2^: 0.126		*R*^2^: 0.099	

PC, Principal component; MDS-UPDRS-III, Movement Disorders Society–Unified Parkinson’s disease Rating Scale III; LED, Levodopa Equivalant Dose. Bold values represent significant contributors to the regression model (*p* < 0.05).

### *Post-hoc* domain specific multiple regression analysis

Additional multiple linear regression analyses were performed to predict cognitive domain *z*-scores for the domains of memory, executive function, attention, language, and visuospatial abilities (Table 2). For the memory domain a significant regression model was found with PC3, PC5, and MDS-UPDRS part III as significant predictors (*R*^2^ = 0.336, *p* < 0.001). The regression model for the prediction of the executive function domain included the MDS-UPDRS part III, PC3, and PC7 as significant predictors (*R*^2^ = 0.312, *p* < 0.001). The attention *z*-score was significantly predicted with a model including PC3, MDS-UPDRS Part III, and PC7 (*R*^2^ = 0.282, *p* < 0.001). For the language domain *z*-score, a significant regression model was found with PC3 and PC7 (*R*^2^ = 0.126, *p* = 0.003). Finally, the regression model predicting visuospatial domain *z*-score included the MDS-UPDRS part III score (*R*^2^ = 0.099, *p* = 0.003).

## Discussion

Our data-driven approach applying a cholinergic PET VOI-based PCA analysis identified seven cholinergic components contributing to brain cholinergic binding variance in PD without dementia. Unlike our *a priori* hypothesis, posterior cortical cholinergic innervation was not a significant predictor of cognition in our patient population. However, the “centro-cingulate” PC, the right-sided fronto-temporal PC and the left sided temporal PC were related to general cognitive function and domain specific *z*-scores for memory, executive function, attention, and language. The centro-cingulate PC was most robustly related to multiple-domain cognitive functioning. These findings, derived by input of a data driven approach, provide confirmation of our recent whole brain regional correlation topographic patterns, that include major components of the centro-cingulate PC identified in the current study. Our findings also provide support for the role of regional and large scale neural cholinergic networks rather than a diffuse neuromodulator system to explain cognitive changes in PD.

### Principal components

The grouping of the PCs seems primarily based on anatomical proximity and cholinergic topographical projections. For example, PC1 and PC4 grouped regions of anatomical proximity of the posterior and frontal parts of the brain, respectively. This may reflect the presence of regional but bilateral cholinergic neural circuits, which may also be the case for PC2, representing predominantly bilateral subcortical regions. Also, the temporal regions showed more hemispheric grouping in PC5 and PC7, which suggests a role of anatomical proximity or the presence of cholinergic lateralizing (dominant vs. non-dominant) hemispheric cognitive circuits in the grouping of PCs.

Rather than driven by regional anatomical neural circuits, it is also possible that the PCA components may reflect (PD-related) vulnerability of cholinergic projections. For example, previous research has demonstrated more prominent posterior cholinergic denervation in PD, at least in early stage disease (Hilker et al., 2005; Shimada et al., 2009; Klein et al., 2010; van der Zee et al., 2019). This comprises the same regions as PC1, including the occipital lobe, parietal lobe, and posterior regions of the temporal lobes. PC1 is also the component with the largest explained variance. Furthermore, the grouping of cortical regions shows overlap with basal forebrain cholinergic projections, in particular projections from the Ch4 group, the nucleus basalis of Meynert (NbM) (Liu et al., 2015). The anteromedial part of the Ch4 (Ch4am) is thought to project to the medial cortical regions, including the cingulate cortex, which shows considerable overlap with PC3. The posterior Ch4 group (Ch4p) projects to the superior temporal and temporal polar regions; partly represented in PC5 (right sided) and PC7 (predominantly left-sided). Neuronal loss within the NbM has repeatedly been demonstrated in PD (Arendt et al., 1983; Gaspar and Gray, 1984; Zarow et al., 2017) and is associated with structural changes of the innervated regions in Alzheimer’s disease (Cantero et al., 2017). Overlap between NbM projections and the cortical cholinergic subcomponents found in our data may therefore indicate a role of PD cholinergic pathology in the grouping of cholinergic PCs.

Our results on the PCA in the HC group substantiate this finding. In contrast to the components in the PD group, the first and largest component (PC1) in the HC group represented a more widespread cortical topography, including the occipital lobe, parietal lobe, central region, and significant regions of the temporal and frontal lobes. This topography shows substantial overlap with cholinergic projections originating from the NbM. However, HC data do not demonstrate the distinctive centro-cingulate component as shown in PD, suggesting either PD specific (basal forebrain) pathology projecting to these regions or intrinsic PD-specific cholinergic vulnerability of large scale neural networks. Furthermore, HC data demonstrated separate PCs for the transverse and superior temporal region, possibly representing Ch4p projections, and the predominantly right sided inferior frontal gyrus. PD components PC5 and PC7, representing right-sided frontal and temporal regions, and left sided temporal regions, respectively, are less prominently present in HC and more distinctive for PD.

Interestingly, the regions distinctive for PD when compared to HC, including PD components PC3, PC5, and PC7, are the three regions related to cognitive functioning in PD. These regions may represent PD-specific cholinergic pathology that plays a significant role in the clinical symptomatology of PD.

### Cognition

Our regression analysis on the relationship between the component scores of the cholinergic covarying patterns and cognitive functioning identified PC3, PC5, and PC7 as significant contributors in addition to motor impairment. These regions are characterized by bilateral centro-cingulate regions (PC3), including the pre-, para-, and post-central gyri, the cingulate cortex (posterior and mid to anterior regions), the right-sided frontal and temporal regions (PC5) and the predominantly left sided medial temporal regions (PC7). PC3, the centro-cingulate region, showed the most robust relationship with both general as well as domain-specific cognitive functioning.

Although derived from a totally different analytic approach, these findings share topographic overlap with our previous study, in which we identified a shared cholinergic topography related to cognitive functioning across domains in a whole brain voxel-based analysis (van der Zee et al., 2020). The previously identified regions included cingulate cortex, the insula and operculum, and the (visual) thalamus. Between the two studies with two different approaches, the cingulate cortex is an area of prominent topographic overlap between these two analyses. The involvement of the cingulate cortex is not surprising. Reduced blood flow in the posterior cingulate cortex is predictive of global cognitive decline rather than being limited to impaired memory functions in Alzheimer’s disease (Huang et al., 2002). In PD, previous research has revealed an association between abnormalities of the posterior cingulate cortex and cognitive impairment in PD, and hypometabolism of the posterior cingulate cortex was predictive for dementia in PD (Bohnen et al., 2011; Leech and Sharp, 2014; Christopher et al., 2015; de Schipper et al., 2017). The current study adds to our previous findings that the cingulate cortex shows a covarying pattern with additional central structures including the pre-, para-, and post-central gyri and the bilateral superior frontal gyri, represented by the PC3. The precentral gyrus, postcentral gyrus, and paracentral lobule have previously been identified as important nodes of an altered subnetwork in elderly with subjective cognitive deficits and shown to be related to cognitive assessment (Kim et al., 2019).

Moreover, the topography of PC3 (i.e., midline structures combined with bilateral peri-central regions) may reflect an important hub region of more large-scale neural networks, spanning anterior and posterior cortical regions. For example, the posterior cingulate cortex and the anterior mesofrontal part of PC3 form key regions of the default mode network (DMN) (Raichle et al., 2001; Ward et al., 2014; Raichle, 2015). As a central region in the DMN, the posterior cingulate cortex has widespread functional connectivity with cortical regions and is associated with multiple cognitive processes including memory, attention and visuospatial abilities (Tessitore et al., 2012; Lee et al., 2018). A decline in functional connectivity of the DMN is correlated with impaired verbal and visual memory performance in PD (Lucas-Jiménez et al., 2016). Our findings of involvement of two of the key hubs of the DMN, suggest that the cholinergic system may be an important contributor to the integrity of the DMN in PD. Furthermore, a graph theory analysis has recently identified the cingulo-opercular and salience functional MRI-defined large-scale networks as major components of the so-called cingulo-opercular task control (COTC) network (Power et al., 2011). The COTC network has been implicated in important cognitive functions, including maintenance of alertness, task set maintenance, and salience detection of stimuli (Dosenbach et al., 2007, 2008; Sadaghiani and D’Esposito, 2015; Coste and Kleinschmidt, 2016). Given the partly overlap with our findings, including the involvement of the cingulate cortex and medial superior frontal cortex, the cholinergic system may play an important role in the COTC network. Interestingly, the preferential cholinergic involvement of the COTC has also been shown in patients with Dementia with Lewy bodies (DLB), in which cognitive fluctuations and attentional deficits are common (Kanel et al., 2020).

In line with our previous voxel-based analysis, we show overlapping topography across multiple cognitive domains, with the exception of the visuospatial domain (van der Zee et al., 2020). The regions include the PC3, PC5, and PC7, representing primarily the centro-cingulate and bilateral temporal regions, of which the PC3 region is most prominently found to be associated with various cognitive functions. Overall, our findings demonstrate a key role of the centro-cingulate region across multiple cognitive domains that may reflect involvement of hubs of distributed neural networks, both on a large scale and more local circuits. Future research combining cholinergic innervation status with neural network analysis is needed to further explore and specify this role.

Another interesting finding was that PC1, reflecting predominantly the occipital lobe, parietal lobe and posterior regions of the temporal lobe, showed no significant correlations with cognitive functioning or other PD related clinical variables, including disease duration, LED, or severity of motor impairment. This is surprising, as multiple previous *in vivo* studies on the cholinergic system in PD demonstrated posterior cholinergic denervation in PD (at least in early stage disease) compared to control subjects (Hilker et al., 2005; Shimada et al., 2009; Klein et al., 2010; van der Zee et al., 2019). A possible explanation could be that (isolated) local posterior changes are not sufficient to cause clinical changes, but that involvement of important neural network hubs, including the centro-cingulate regions, may cause more large-scale cholinergic disruptions underlying cognitive impairment in PD. Furthermore, our observations may reflect the importance of the predominantly anterior brain dopaminergic system innervation, which may dynamically interact with cholinergic changes underlying cognitive impairment. Dopaminergic frontal denervation has also been shown to induce posterior cortical thinning (Sampedro et al., 2019), suggesting again a possible role of large-scale network dysfunction in the cognitive impairment syndrome in PD. An alternative hypothesis is that given the more prominent glucose metabolic or blood flow changes in the posterior cortices may reflect a more global neurodegenerative process rather than intrinsic vulnerability of the cholinergic system *per se*.

### Strengths and limitations

An important strength of this study is the data-driven approach to identify covarying cholinergic brain regions to test associations with cognition rather than *a priori* selected brain regions. This data-driven approach of the PCA is of additional value to the previously published voxel-based analysis (van der Zee et al., 2020). Although the voxel-based analysis allows a detailed evaluation of regional specific correlations, the current evaluation explains more of the cholinergic brain functioning as a whole, instead of region specific. The previously identified cholinergic regions involved in cognitive functioning are now shown to most likely be part of larger scale networks underlying cognitive performance. We selected a VOI-based approach where results may be more straightforward to interpret patterns compared to voxel-based methods that may identify similar patterns separately. Another strength is the use of cognitive *z*-scores for the evaluation of cognitive functioning. *Z*-scores are cognitive scores already corrected for age, gender, and educational level, eliminating these variables as possible covarying components.

A limitation of the current study is the cross-sectional nature that precludes effects of progressive cholinergic innervation changes and interval changes in cognition. Another limitation is the smaller sample size for the HC group compared to the PD group. The smaller HC group and the expected lower heterogeneity of cholinergic binding in het HC group may cause differences in PCA outcome between groups. The final outcome of a factor analysis is influenced by the variables and subjects included. Our findings therefore need to be substantiated by future multivariate analysis studies on the relationship between cognition and cholinergic innervation, preferably including PD patients at different disease stages and cognitive status.

### Conclusion

The results of this data-driven approach demonstrate a centro-cingulate covarying cholinergic pattern as a key predictor of cognitive impairment in PD, in both general cognitive functioning as well as domain specific cognition for memory, attention, executive function, and language domains. Our findings also provide support for the role of regional and large scale neural cholinergic networks rather than a diffuse neuromodulator system to explain cognitive changes in PD.

## Data availability statement

The datasets presented in this article are not readily available because of data sharing restrictions as some participants are US Veterans. Requests to access the datasets should be directed to NB and will be considered upon reasonable request for non-veteran participants.

## Ethics statement

The studies involving human participants were reviewed and approved by Institutional Review Boards of the University of Michigan School of Medicine and Veterans Affairs Ann Arbor Healthcare System. The patients/participants provided their written informed consent to participate in this study.

## Author contributions

SZ: design and execution of the statistical analysis, writing the first draft of the manuscript, and review and critique of additional versions. PK: execution of the research project, execution and review of the statistical analysis, and review and critique of the manuscript. MM and NB: conception, organization, and execution of the research project, design and review of the statistical analysis, and review and critique of the manuscript. TL: review and critique of the statistical analysis and manuscript. All authors contributed to the article and approved the submitted version.

## Footnotes

1
http://surfer.nmr.mgh.harvard.edu
