# Supplementary material for: Identification of cholinergic centro-cingulate topography as main contributor to cognitive functioning in Parkinson’s disease: Results from a data-driven approach
Source: Front Aging Neurosci. 2022 Oct 20;14:1006567. doi: 10.3389/fnagi.2022.1006567 (PMC9631831; doi:10.3389/fnagi.2022.1006567)
Supplement: Supplementary file 1 [file Data_Sheet_1.DOCX]

## *Supplementary material*


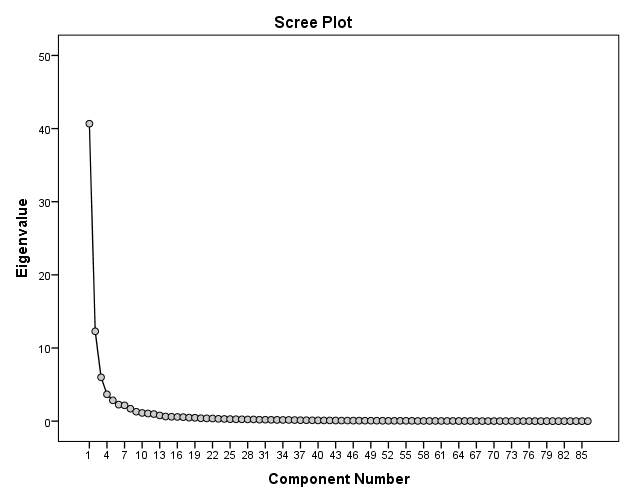
Supplementary figure 1: Screeplot showing the eigenvalues of each

component of the principal component analysis in PD subjects.

| **Supplementary table 1: Loading factors for each of the seven main principal components in PD** | | | | | | | |
| --- | --- | --- | --- | --- | --- | --- | --- |
|  | Components | | | | | |  |
|  | 1 | 2 | 3 | 4 | 5 | 6 | 7 |
| pericalcarine_R | 0.909 |  |  |  |  |  |  |
| pericalcarine_L | 0.907 |  |  |  |  |  |  |
| cuneus_L | 0.906 |  |  |  |  |  |  |
| inf_par_L | 0.900 |  |  |  |  |  |  |
| inf_par_R | 0.887 |  |  |  |  |  |  |
| cuneus_R | 0.885 |  |  |  |  |  |  |
| lingual_L | 0.847 |  |  |  |  |  |  |
| lingual_R | 0.840 |  |  |  |  |  |  |
| lat_occ_L | 0.839 |  |  |  |  |  |  |
| lat_occ_R | 0.818 |  |  |  |  |  |  |
| sup_par_R | 0.816 |  |  |  |  |  |  |
| sup_par_L | 0.811 |  |  |  |  |  |  |
| precuneus_L | 0.773 |  | 0.512 |  |  |  |  |
| precuneus_R | 0.769 |  | 0.463 |  |  |  |  |
| ctx_bankssts_L | 0.739 |  |  |  |  |  |  |
| supramarg_L | 0.719 |  |  |  |  |  |  |
| ctx_bankssts_R | 0.707 |  |  |  | 0.482 |  |  |
| mid_temp_L | 0.694 |  |  |  |  |  |  |
| fusiform_L | 0.680 |  |  |  |  |  | 0.460 |
| inf_temp_L | 0.664 |  |  |  |  |  | 0.496 |
| supramarg_R | 0.631 |  |  |  | 0.557 |  |  |
| mid_temp_R | 0.629 |  |  |  | 0.551 |  |  |
| fusiform_R | 0.621 |  |  |  | 0.565 |  |  |
| sup_temp_L | 0.588 |  |  |  |  |  |  |
| transv_temp_L | 0.581 |  |  |  |  |  |  |
| sup_temp_R | 0.564 |  |  |  | 0.564 |  |  |
| transv_temp_R | 0.547 |  |  |  | 0.430 |  |  |
| caud_mid_fro_L | 0.476 |  | 0.445 |  |  |  |  |
| Putamen_R |  | 0.950 |  |  |  |  |  |
| Putamen_L |  | 0.947 |  |  |  |  |  |
| Caudate_R |  | 0.856 |  |  |  |  |  |
| Caudate_L |  | 0.835 |  |  |  |  |  |
| Accumbens_L |  | 0.795 |  |  |  |  |  |
| Accumbens_R |  | 0.748 |  |  |  |  |  |
| Amygdala_L |  | 0.730 |  |  |  |  |  |
| Pallidum_L |  | 0.711 |  |  |  |  |  |
| Thalamus_L |  | 0.711 |  |  |  |  |  |
| Thalamus_R |  | 0.694 |  |  |  |  |  |
| Amygdala_R |  | 0.691 |  |  |  |  |  |
| Hippocampus_R |  | 0.676 |  |  |  |  |  |
| Hippocampus_L |  | 0.675 |  |  |  |  |  |
| Pallidum_R |  | 0.649 |  |  |  |  |  |
| insula_R |  | 0.504 |  |  | 0.418 |  |  |
| insula_L |  | 0.458 |  | 0.452 |  |  |  |
| paracentral_L |  |  | 0.838 |  |  |  |  |
| post_cing_L |  |  | 0.823 |  |  |  |  |
| post_cing_R |  |  | 0.816 |  |  |  |  |
| paracentral_R |  |  | 0.815 |  |  |  |  |
| isthmus_cing_R |  |  | 0.713 |  |  |  |  |
| isthmus_cing_L |  |  | 0.674 |  |  |  |  |
| precentral_L | 0.401 |  | 0.632 |  |  |  |  |
| caud_ant_cing_L |  |  | 0.606 |  |  |  |  |
| precentral_R |  |  | 0.589 |  | 0.516 |  |  |
| sup_front_L |  |  | 0.578 |  |  |  |  |
| sup_front_R |  |  | 0.566 |  | 0.469 |  |  |
| postcentral_L | 0.542 |  | 0.547 |  |  |  |  |
| frontalpole_L |  |  |  | 0.829 |  |  |  |
| parsorbitalis_L |  |  |  | 0.757 |  |  |  |
| parsorbitalis_R |  |  |  | 0.723 |  |  |  |
| parstriangularis_L |  |  |  | 0.690 |  |  |  |
| frontalpole_R |  |  |  | 0.687 |  |  |  |
| lat_orb_fro_L |  |  |  | 0.649 |  |  |  |
| lat_orb_fro_R |  |  |  | 0.618 |  |  |  |
| med_orb_fro_L |  |  |  | 0.566 |  |  |  |
| rostr_mid_front_L | 0.449 |  |  | 0.532 |  |  |  |
| med_orb_fro_R |  | 0.404 |  | 0.496 | 0.407 |  |  |
| temp_pole_R |  |  |  |  |  |  |  |
| parsopercularis_R |  |  |  |  | 0.681 |  |  |
| parstriangularis_R |  |  |  | 0.471 | 0.658 |  |  |
| inf_temp_R | 0.563 |  |  |  | 0.625 |  |  |
| rostr_mid_front_R | 0.408 |  |  | 0.435 | 0.606 |  |  |
| parahipp_R |  |  |  |  | 0.595 |  |  |
| caud_mid_fro_R | 0.511 |  |  |  | 0.594 |  |  |
| postcentral_R | 0.471 |  | 0.508 |  | 0.555 |  |  |
| CBL_GM_L |  |  |  |  |  | 0.907 |  |
| CBL_GM_R |  |  |  |  |  | 0.897 |  |
| CBL_WM_R |  |  |  |  |  | 0.897 |  |
| CBL_WM_L |  |  |  |  |  | 0.888 |  |
| entorhinal_L |  |  |  |  |  |  | 0.718 |
| temp_pole_L |  |  |  |  |  |  | 0.569 |
| parahipp_L |  |  |  |  |  |  | 0.536 |
| entorhinal_R |  |  | 0.408 |  |  |  | 0.487 |
| parsopercularis_L |  |  |  | 0.441 |  |  |  |
| rostr_ant_cing_L |  |  |  |  |  |  |  |
| rostr_ant_cing_R |  |  |  |  | 0.414 |  |  |
| caud_ant_cing_R |  |  |  |  |  |  |  |


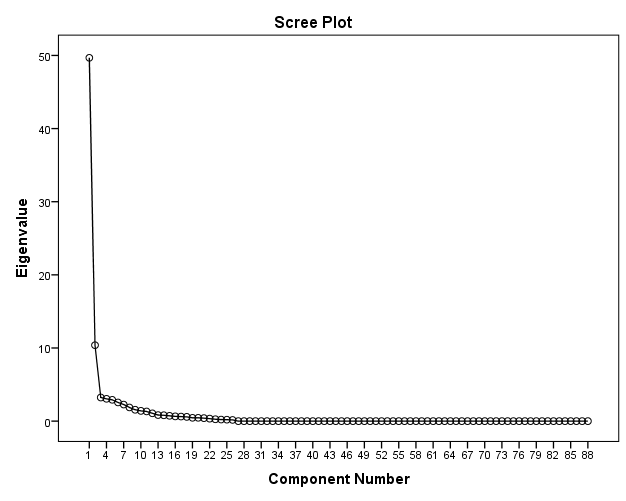


Supplementary figure 2: screeplot showing the eigenvalues of each component of

the principal component analysis in HC subjects.

| **Supplementary table 2: Loading factors for each of the seven main principal components of healthy controls** | | | | | | | |
| --- | --- | --- | --- | --- | --- | --- | --- |
|  | **Component** | | | | | | |
| Region | 1 | 2 | 3 | 4 | 5 | 6 | 7 |
| ctx-rh-superiorparietal | 0.938 |  |  |  |  |  |  |
| ctx-rh-inferiorparietal | 0.914 |  |  |  |  |  |  |
| ctx-lh-superiorparietal | 0.913 |  |  |  |  |  |  |
| ctx-lh-lateraloccipital | 0.894 |  |  |  |  |  |  |
| ctx-lh-inferiorparietal | 0.894 |  |  |  |  |  |  |
| ctx-rh-precuneus | 0.883 |  |  |  |  |  |  |
| ctx-lh-lingual | 0.85 |  |  |  |  |  |  |
| ctx-rh-lateraloccipital | 0.849 |  |  |  |  |  |  |
| ctx-lh-pericalcarine | 0.831 |  |  |  |  |  |  |
| ctx-lh-cuneus | 0.826 |  |  |  |  |  |  |
| ctx-lh-precuneus | 0.81 |  |  |  |  |  |  |
| ctx-rh-lingual | 0.738 |  |  |  |  |  |  |
| ctx-rh-postcentral | 0.738 |  |  |  |  |  |  |
| ctx-rh-pericalcarine | 0.737 |  |  |  |  |  |  |
| ctx-rh-cuneus | 0.726 |  |  |  |  |  |  |
| ctx-rh-supramarginal | 0.724 |  |  |  |  | 0.405 |  |
| ctx-lh-paracentral | 0.703 |  |  |  |  |  |  |
| ctx-rh-superiorfrontal | 0.698 |  |  |  |  |  |  |
| ctx-lh-bankssts | 0.694 |  |  |  |  |  |  |
| ctx-rh-bankssts | 0.694 |  |  |  |  | 0.432 |  |
| ctx-lh-inferiortemporal | 0.685 |  |  |  |  |  |  |
| ctx-lh-fusiform | 0.681 |  |  |  |  |  |  |
| ctx-lh-postcentral | 0.674 |  |  |  |  |  |  |
| ctx-lh-supramarginal | 0.66 |  |  |  |  | 0.465 |  |
| ctx-rh-fusiform | 0.656 |  |  |  |  |  |  |
| ctx-lh-superiorfrontal | 0.649 |  |  |  |  |  |  |
| ctx-rh-precentral | 0.642 |  |  | 0.403 |  |  |  |
| ctx-lh-posteriorcingulate | 0.626 |  | 0.524 |  |  |  |  |
| ctx-rh-middletemporal | 0.623 |  |  | 0.441 |  |  |  |
| ctx-lh-middletemporal | 0.614 |  |  |  |  |  |  |
| ctx-lh-precentral | 0.606 |  |  |  |  |  |  |
| ctx-lh-parahippocampal | 0.604 |  |  |  |  |  |  |
| ctx-rh-paracentral | 0.595 |  | 0.474 |  |  |  |  |
| ctx-rh-caudalmiddlefrontal | 0.587 |  |  | 0.551 |  |  |  |
| ctx-rh-inferiortemporal | 0.559 |  |  | 0.419 |  |  |  |
| ctx-lh-isthmuscingulate | 0.537 |  | 0.463 |  |  |  |  |
| ctx-lh-rostralmiddlefrontal | 0.511 |  |  | 0.447 |  |  |  |
| ctx-lh-parsopercularis | 0.508 |  |  |  |  |  |  |
| ctx-rh-isthmuscingulate | 0.491 |  |  |  |  |  |  |
| ctx-lh-caudalmiddlefrontal | 0.476 |  |  |  |  |  |  |
| Left-Putamen |  | 0.969 |  |  |  |  |  |
| Right-Putamen |  | 0.962 |  |  |  |  |  |
| Right-Accumbens-area |  | 0.932 |  |  |  |  |  |
| Left-Amygdala |  | 0.916 |  |  |  |  |  |
| Right-Caudate |  | 0.87 |  |  |  |  |  |
| Left-Pallidum |  | 0.856 |  |  |  |  |  |
| Left-Accumbens-area |  | 0.821 |  |  |  |  |  |
| Left-Caudate |  | 0.818 |  |  |  |  |  |
| Right-Amygdala |  | 0.814 |  |  |  |  |  |
| Left-VentralDC |  | 0.798 |  |  |  |  |  |
| Right-VentralDC |  | 0.73 |  |  |  |  |  |
| Right-Pallidum |  | 0.655 |  |  |  |  |  |
| Left-Hippocampus | 0.404 | 0.65 |  |  |  |  |  |
| ctx-lh-insula |  | 0.644 |  |  |  |  |  |
| ctx-rh-temporalpole |  | 0.64 |  |  |  |  |  |
| Left-Thalamus-Proper |  | 0.63 |  |  |  |  |  |
| Right-Hippocampus | 0.412 | 0.627 |  |  |  |  |  |
| Right-Thalamus-Proper |  | 0.594 |  |  |  |  |  |
| ctx-lh-entorhinal |  | 0.594 |  |  |  |  |  |
| ctx-rh-lateralorbitofrontal |  | 0.529 |  | 0.401 |  |  | 0.47 |
| ctx-rh-rostralanteriorcingulate | | 0.516 | 0.516 |  |  |  |  |
| ctx-rh-insula |  | 0.508 |  |  |  |  |  |
| ctx-lh-parstriangularis |  | 0.491 |  | 0.435 |  |  |  |
| ctx-rh-caudalanteriorcingulate | |  | 0.711 |  |  |  |  |
| ctx-lh-rostralanteriorcingulate | |  | 0.708 |  |  |  |  |
| ctx-lh-caudalanteriorcingulate | |  | 0.7 |  |  |  |  |
| ctx-rh-posteriorcingulate | 0.577 |  | 0.654 |  |  |  |  |
| ctx-rh-medialorbitofrontal | 0.419 |  | 0.46 |  |  |  | 0.448 |
| ctx-rh-parstriangularis |  |  |  | 0.747 |  |  |  |
| ctx-rh-parsopercularis | 0.409 |  |  | 0.669 |  |  |  |
| ctx-rh-rostralmiddlefrontal | 0.587 |  |  | 0.657 |  |  |  |
| ctx-rh-parsorbitalis |  |  |  | 0.64 |  |  |  |
| ctx-lh-parsorbitalis | 0.424 |  |  | 0.428 | 0.409 |  |  |
| Left-Cerebellum-Cortex |  |  |  |  | 0.821 |  |  |
| Right-Cerebellum-Cortex | 0.406 |  |  |  | 0.796 |  |  |
| Left-Cerebellum-White-Matter | |  |  |  | 0.794 |  |  |
| Right-Cerebellum-White-Matter | |  |  |  | 0.788 |  |  |
| ctx-lh-transversetemporal | 0.472 |  |  |  |  | 0.791 |  |
| ctx-rh-transversetemporal | 0.422 |  |  |  |  | 0.694 |  |
| ctx-rh-superiortemporal | 0.505 |  |  |  |  | 0.576 |  |
| ctx-lh-superiortemporal | 0.546 |  |  |  |  | 0.566 |  |
| ctx-lh-frontalpole |  |  |  |  |  |  | 0.81 |
| ctx-rh-frontalpole |  |  |  |  |  |  | 0.623 |
| ctx-lh-medialorbitofrontal |  |  |  |  |  |  | 0.563 |
| ctx-lh-lateralorbitofrontal | 0.403 | 0.41 |  |  |  |  | 0.486 |
| ctx-rh-parahippocampal | 0.521 |  |  |  |  |  |  |
| ctx-rh-entorhinal |  | 0.54 |  |  |  |  |  |
| ctx-lh-temporalpole |  | 0.513 |  |  |  |  |  |
